# Evidence for Biotrophic Lifestyle and Biocontrol Potential of Dark Septate Endophyte *Harpophora oryzae* to Rice Blast Disease

**DOI:** 10.1371/journal.pone.0061332

**Published:** 2013-04-18

**Authors:** Zhen-Zhu Su, Li-Juan Mao, Na Li, Xiao-Xiao Feng, Zhi-Lin Yuan, Li-Wei Wang, Fu-Cheng Lin, Chu-Long Zhang

**Affiliations:** 1 State Key Laboratory for Rice Biology, Institute of Biotechnology, Zhejiang University, Hangzhou, China; 2 Analysis Center of Agrobiology and Environmental Science, Zhejiang University, Hangzhou, China; 3 Institute of Subtropical Forestry, Chinese Academy of Forestry, Fuyang, Zhejiang Province, China; 4 Department of Pharmaceutical Science, School of Health Management, Hangzhou Normal University, Hangzhou, China; 5 China Tobacco Gene Research Center, Zhengzhou Tobacco Research Institute of CNTC, Zhenzhou, China; Seoul National University, Republic of Korea

## Abstract

The mutualism pattern of the dark septate endophyte (DSE) *Harpophora oryzae* in rice roots and its biocontrol potential in rice blast disease caused by *Magnaporthe oryzae* were investigated. Fluorescent protein-expressing *H. oryzae* was used to monitor the colonization pattern. Hyphae invaded from the epidermis to the inner cortex, but not into the root stele. Fungal colonization increased with root tissue maturation, showing no colonization in the meristematic zone, slight colonization in the elongation zone, and heavy colonization in the differentiation zone. *H. oryzae* adopted a biotrophic lifestyle in roots accompanied by programmed cell death. Real-time PCR facilitated the accurate quantification of fungal growth and the respective plant response. The biocontrol potential of *H. oryzae* was visualized by inoculation with eGFP-tagged *M. oryzae* in rice. *H. oryzae* protected rice from *M. oryzae* root invasion by the accumulation of H_2_O_2_ and elevated antioxidative capacity. *H. oryzae* also induced systemic resistance against rice blast. This systemic resistance was mediated by the *OsWRKY45*-dependent salicylic acid (SA) signaling pathway, as indicated by the strongly upregulated expression of *OsWRKY45*. The colonization pattern of *H. oryzae* was consistent with the typical characteristics of DSEs. *H. oryzae* enhanced local resistance by reactive oxygen species (ROS) and high antioxidative level and induced *OsWRKY45*-dependent SA-mediated systemic resistance against rice blast.

## Introduction

Plants are often symbiotically associated with a wide range of both prokaryotic and eukaryotic microbes. Some plant traits, including growth, metabolism, and stress adaptation mediated by these beneficial microorganisms, show great promise in the commercial development of microbial agents for agricultural sustainability [1].

Fungal endophytes, an important component of plant-associated mycobionts, have attracted a great deal of attention due to their striking species and functional diversity. Currently, four classes of endophytes have been defined based on a series of criteria. The ecology and chemistry (alkaloids) of grass clavicipitaceous Class 1 endophytes has been extensively documented over the past several decades. Class 3 endophytes, often with a localized infection pattern in aboveground tissues, are the best-studied group and are known for their extremely high species and chemical diversity [2]. In comparison, Class 2 and 4 endophytes are poorly characterized. Rodriguez *et al.* [3] and Redman *et al.* [4] proposed that plant stress tolerance would be enhanced through symbiosis with Class 2 endophytes in a habitat-specific manner. This mode of action is often simultaneously accompanied with extensive colonization by endophytes in whole plants but with less species diversity. Furthermore, Class 2 endophytes may be found only in stressful environments but may be less ubiquitous in natural ecosystems in comparison with Class 3 and 4 endophytes.

Only Class 4 endophytes preferentially infect plant roots. Dark septate endophytes (DSEs) are representative Class 4 endophytes characterized by melanized hyphae and microsclerotia formed in cortical cells [5]–[7]. More than 600 plant species in 114 families with a wide geographic distribution have been shown to harbor DSEs. In some cases, DSEs are predominant compared to mycorrhizal fungi, indicating that they represent an equally important root fungal community [8]. It is generally accepted that DSEs comprise a polyphyletic group of ascomycetes [9], [10]. The *Gaeumannomyces-Phialophora* complex and *Phialocephala fortinii-Acephala applanata* species complex constitute the best-studied major subgroups of DSEs [9]. Certain members of the *Phialophora*, now assigned to the genus *Harpophora*, usually live in herbaceous plant roots as hosts, especially in Poaceae.

However, the biological effects of DSEs on plant growth and fitness remain elusive. Similar to other groups of plant-associated microbes, the outcomes of root-DSEs interactions are variable, ranging from mutually beneficial to neutral to harmful [9], [11]–[16]. Despite the ecological significance of DSEs, no information is available regarding the deep morphological and molecular aspects of this type of symbiosis.

During the investigation of endophytes residing in domestic Chinese wild rice (*Oryza granulata*), the occurrence of DSEs was determined using microscopic and culture-based approaches and a novel species named *H. oryzae* was described. *In vitro* pathogenicity tests showed that *H. oryzae* acted as a nonpathogenic endophyte colonizing cultivated rice (*Oryza sativa* L.) roots, and the melanized hyphae colonized the root surface and cortical cells intracellularly [17]. Similar to *H. graminicola*, a beneficial DSE in grasses [12], [18], *H. oryzae* strongly promotes rice growth and biomass increases. It is likely that the DSEs in *Harpophora* are true, beneficial endophytes.

ROS and antioxidants, which are universal and evolutionarily conserved, are likely to play important roles in symbiotic interactions. ROS play a vital role in programmed cell death (PCD), stress responses, plant defenses against pathogens, and systemic stress signaling in conjunction with antioxidant production [19]. Fungus-derived ROS play a critical role in *Epichloë festucae*-*Lolium perenne* mutualistic symbiosis, and dominates fungal embranchment and the biomass inside host leaves [20]. Antioxidants are the means by which ROS are mediated and regulated to avoid or reduce cell damage and death. These include nonenzymic antioxidants such as ascorbic acid (AsA), glutathione (GSH), phenolics, carotenoids, and tocopherols as well as enzymic antioxidants, including superoxide dismutase (SOD), catalase (CAT), peroxidase (POD), ascorbate peroxidase, GSH reductase (GR), monodehydroascorbate reductase (MDHAR) and dehydroascorbate reductase (DHAR). The increases in antioxidant levels are correlated with increased plant tolerance to multiple stresses. A delicate equilibrium between ROS production and scavenging is important to maintain plant-fungus interactions. However, there have been few studies of the potential role of ROS and/or antioxidant activity in endophyte-mediated plant resistance to pathogens.

Rice is a staple food for half of the global population, and rice blast caused by *M. oryzae* is a constant threat to the world’s food supply. Control strategies depend on the use of resistant cultivars and the application of fungicides, although neither of these methods is particularly effective [21]. Therefore, the development of durable, environmentally secure strategies for the biocontrol of rice blast disease is urgently required. A better understanding of the *Harpophora* lifestyle and biological behavior will provide effective and novel means for the management of this disease.

The present study was performed to monitor the distribution and colonization pattern of *H. oryzae* in root tissues through the expression of the fluorescent protein DsRed2 or eGFP as a marker in mycelia. Real-time PCR was also used to accurately quantify the growth and development of *H. oryzae*. In addition, we examined whether *H. oryzae* has the potential to confer disease resistance to rice cultivars and the underlying molecular mechanism of any such interaction. Our findings will enhance our understanding of the mutualistic interaction between *H. oryzae* and rice. With regard to biotechnological applications, it may be possible to transfer native DSEs to non-native plants to enhance crop production.

## Materials and Methods

### Endophytic and Pathogenic Fungi, Bacterial Strains and Plant Materials

Two endophytic *H. oryzae* strains, R5-6-1 and RC-3-1, were isolated from healthy wild rice roots [17] and cultured on complete medium agar (CM) [22] at 25°C in the dark. The eGFP-tagged *M. oryzae* strain Guy11 (wild-type, MAT1-2) [23], [24] was used as a pathogen.

*Agrobacterium tumefaciens* strain AGL-1 [25] carrying the plasmid pKD6-GFP or pKD5-RED [26] was used for the genetic transformation of *H. oryzae*.

The blast-susceptible rice cultivar CO-39 (*O. sativa*) was used as a compatible host plant for inoculation experiments.

### The *A. tumefaciens*-mediated Transformation of *H. oryzae*

pKD5-RED containing the sulfonylurea resistance gene and DsRed2 gene under the control of the exogenous histone H3 promoter and ribosomal protein 27 terminator on the backbone of modified pCAMBIA 1300 (Cambia, Brisbane, Qld, Australia) [27] was introduced into *A. tumefaciens* strain AGL-1 and transformed into *H. oryzae* as described [28]. A second vector, pKD6-GFP, carried eGFP gene which is identical to pKD5-RED except it contains the SOD1 promoter instead of the H3 promoter [26]. The fluorescence expression of the transformants was assessed by an LSM780 laser scanning confocal microscope (Carl Zeiss Inc., Jena, Germany). eGFP and DsRed fluorescence were excited with 488 and 561 nm lasers, and detected at 500–530 and 560–600 nm, respectively.

### Co-culture, Root Staining and Microscopy

Rice seeds without capsules were surface-sterilized in 70% ethanol for 8 min, in 1.0% sodium hypochlorite solution (5% active chlorine) for 10 min, rinsed repeatedly using sterile water, and planted in half-strength Murashige & Skoog (1/2 MS) solid medium for 5 days. The seedlings were then transferred to 1/2 MS in sealed glass tubes (3.6 cm in width, 50 cm in length) and inoculated with *H. oryzae* spores harvested from 3-day-old liquid CM cultures. Each tube of three seedlings received a total of 10^7^ conidia on top of the root system or mock-inoculation as controls (18 tubes each). The plants were kept at 24/22°C with a 16-h-light/8-h-dark photoperiod. The plant roots were sampled and processed for microscopy. The fungal structures were observed under an Olympus fluorescence microscope BX51 (Tokyo, Japan). The root infection process was monitored using an LSM780 laser scanning confocal microscope (Carl Zeiss Inc., Jena, Germany). eGFP fluorescence was excited with 488 nm laser, and detected at 500–530 nm. Root cell wall autofluorescence was detected at 650–700 nm.

To test the viability of infected root cells hosting fungal structures, lipophilic endocytic dye FM4-64 and DAPI were used to monitor endocytosis and endosome formation and cell nuclei, respectively [29], [30].

To distinguish the red fluorescence from endosomes stained with FM4-64, Ho31gfp was used as the inoculum. Inoculated roots were sampled at 10 and 15 days after inoculation (d.a.i.) and treated with the endocytotic lipophilic tracker FM4-64 (10 µM; Invitrogen, Carlsbad, CA) to label membranes for 30 min [29]. Fluorescence of FM4-64 was detected at 640–700 nm using an excitation wavelength of 514 nm.

For DAPI staining, inoculated roots were sampled at 5, 15 and 20 d.a.i. and stained with 1 µg/mL DAPI for 30 min as described [30]. DAPI fluorescence was detected using an Olympus BX51 microscope.

For the detection of H_2_O_2_, infected roots were sampled at 3, 15 and 20 d.a.i., incubated with 1 mg/mL 3,3'-diaminobenzidine (DAB, D-8001; Sigma, St. Louis, MO) (pH 3.8) at room temperature for 8 h, washed in clearing solution (ethanol:acetic acid = 94∶4, v/v) for 1 h [31], and observed under an Olympus BX51 microscope.

### Quantification of *H. oryzae* Biomass in Roots by Real-time PCR

#### Design of specific primers

The ratio of fungus to plant DNA (fungus/plant DNA ratio, FPDR) was used to monitor the fungal infection in rice roots [30]. This ratio was representative of the behavior of the two partners and an accurate mean of obtaining an integrated view of the outcome of the interaction. The degree of fungal progression was determined by the 2^–ΔCt^ method [32], where ΔCt is the difference between the raw cycle threshold (Ct) values of the *H. oryzae tef-1α* gene and rice *Ubiquitin* gene [30], [33]. Specific primers were designed based on *H. oryzae tef-1α*. This gene was partially amplified with the primers EF728 and EF986 using the following profile: 94°C for 3 min followed by 35 cycles of 94°C for 30 s, 52°C for 30 s, and 72°C for 30 s, with a final extension at 72°C for 10 min. The amplified product was then sequenced. The sequence from R5-6-1 was entered into GenBank and obtained an accession number JN857963. This sequence was queried using the BLAST tool at GenBank and aligned manually with the resulting database-matched sequences using the Clustal W multiple alignment tool [34]. The specific primer set SR-F/SR-R (Table S1) was designed to amplify a region of 151 bp.

#### Genomic extraction and real-time PCR

*H. oryzae*-inoculated roots were harvested at 1, 3, 5, 7, 10, 15, 20, 25 and 30 d.a.i. and surface-sterilized to remove all superficial hyphae as described [35]. The roots were then ground in liquid nitrogen. Genomic DNA was extracted from 100 mg of root powder with a Plant DNeasy Kit (Qiagen, Hilden, Germany) according to the manufacturer’s instructions. For real-time PCR, 10 ng of total DNA was amplified in a total volume of 25 µL containing 12.5 µL of 2× SYBR Premix Ex Taq™ (Takara Bio Inc., Shiga, Japan) and 0.25 µL of 25 µM SR-F/SR-R (or OsUbiq-F/R for the rice *Ubiquitin* gene; Table S1) on a Mastercycler ep realplex Thermal Cycler (Eppendorf, Hauppauge, NY). A melting curve analysis was performed to ensure that only a single product was obtained. Ct values were determined using the Realplex Software 2.2.10.84 supplied with the instrument. Each reaction was performed in triplicate. Quantification of the fungal DNA from the root extracts was performed at least twice and the same results were obtained each time.

### Pathogen Infection

For leaf infection using plants at the three-leaf stage (about 15 d.a.i.), each tube was inoculated by spraying 1 mL of a Guy11 conidial suspension (1×10^5^ conidia/mL) containing 0.1% Tween 20 onto the leaves using an airbrush. In another experiment, roots were inoculated with 2 mL of a Guy11 conidial suspension (1×10^5^ conidia/mL) for root infection. The disease severity was assessed as described [36] at 6 days after leaf inoculation and 8 days after root inoculation. The diseased leaf area percentage (%DLA) was recorded to permit a more accurate evaluation of the disease area and severity [31]. The percentages of disease severity and area were determined for at least 50 plants per replicate in at least three independent experiments.

### Biochemical Determination

*H. oryzae*- and mock-inoculated root samples were collected at 15 d.a.i. For enzyme measurements, samples of frozen roots (0.3 g) were homogenized at 4°C in an ice-chilled mortar with liquid nitrogen in QB buffer (100 mM potassium phosphate buffer, pH 7.8, 1 mM EDTA, 1% Triton X-100 and 15% glycerol) [37] without DTT (for the SOD, CAT and POD assays) and with 50 mg of polyvinylpyrrolidone (PVP) per gram of tissue (for the GR assay). Crude homogenates were centrifuged at 15000 × *g* for 15 min at 4°C, and the supernatant fractions were stored at –20°C. The protein content was determined by the Bradford method using BSA as a standard [38].

SOD activity was monitored as described previously [39]. Enzyme activity was calculated by monitoring the reaction mixture for 120 s (at 60 s intervals) at 560 nm by spectrophotometry. CAT activity was assayed by measuring the initial rate of H_2_O_2_ disappearance at 240 nm by spectrophotometry [40]. POD activity was assayed by monitoring guaiacol at 470 nm by spectrophotometry [41]. GR activity was determined by monitoring the oxidation of NADPH at 340 nm with a molar absorption coefficient of 6.2 mM^−1^ cm^−1^ as described [42]. Ascorbate was determined using the bipyridyl method [43]. DHAR activity was assayed spectrophotometrically at 265 nm as reduced GSH-dependent dehydroascorbate oxidation [44]. The assay mixture contained 50 mM sodium phosphate buffer (pH 6.5), 0.1 mM Na_2_EDTA, 20 µM dehydroascorbate, 50 µM GSH, and 20 to 100 µL of extract in a total volume of 2.3 mL. The total GSH concentrations (GSH and oxidized GSH) were measured as described [45]. Each reaction was performed in triplicate. This experiment was performed three times and the same results were obtained each time.

### RNA Isolation and Expression Analysis

Total RNA was extracted from the third leaves of mock- and *H. oryzae*-inoculated plants using TRIzol reagent (Invitrogen) according to the manufacturer’s instructions. First-strand cDNA was synthesized from 5 µg of total RNA using SuperScript II Reverse Transcriptase (Invitrogen Life Technologies, Carlsbad, CA). The absence of contaminating genomic DNA was confirmed by control PCR on RNA that had not been reverse transcribed. To determine which defense pathway was activated by *H. oryzae* during root invasion, the expression of well-established foliar defense genes was examined. We chose six pathogenesis-related (PR) genes (*PR1a*, *PR1b*, *PR2*, *PR4*, *PBZ1* and *PR10b*), three members of the WRKY family (*OsWRKY45*, *OsWRKY53* and *OsWRKY71*), the chitin receptor *CEBiP*, the Ser/Thr protein kinase *ORK10*, the hypersensitive response-associated transcriptional activator *NAC4*, an endochitinase *Chit1*, the elicitor-inducible shikimate kinase *SK2*, a key regulator of salicylic acid (SA)-mediated resistance *NH1* and the jasmonic acid (JA)-inducible *myb* transcription factor *JAmyb*. The sequences of the primers used for qRT-PCR are listed in Supplement Table 1. The constitutively expressed *Actin* gene was used as an endogenous control. The relative gene expression levels were calculated using the 2^–ΔΔCt^ method [32], [46]. Quantitative RT-PCR was conducted at least twice with three replicates from independent biological experiments.

**Table 1:** The activities of antioxidative system in *H. oryzae*-infected rice roots.

	SODU(mg protein) ^−1^	CATU(mg protein) ^−1^	PODU(mg protein) ^−1^	GRU(mg protein) ^−1^	DHARU(mg protein) ^−1^	AsAµmol g^−1^	Total GSHµmol g^−1^
**CK**	1.24±0.10	0.10±0.01	0.09±0.01	1.74±0.11	1.69±0.06	32.81±2.30	3.12±0.11
**R5-6-1**	14.78±0.95**	5.24±0.16**	3.83±0.34**	3.78±0.17**	4.49±0.20**	49.23±1.87*	5.24±0.16**
**Fold**	12.0	52.1	39.8	2.2	2.7	1.5	2.0

Effects of *H. oryzae* on antioxidant activity in rice roots. The values are the means of three samples. Similar results were obtained in three independent experiments. Significance (Student’s *t*-test):

* , *P*<0.05;

** , *P*<0.01.

## Results

### Genetic Transformation of *H. oryzae*

**Figure 1:**
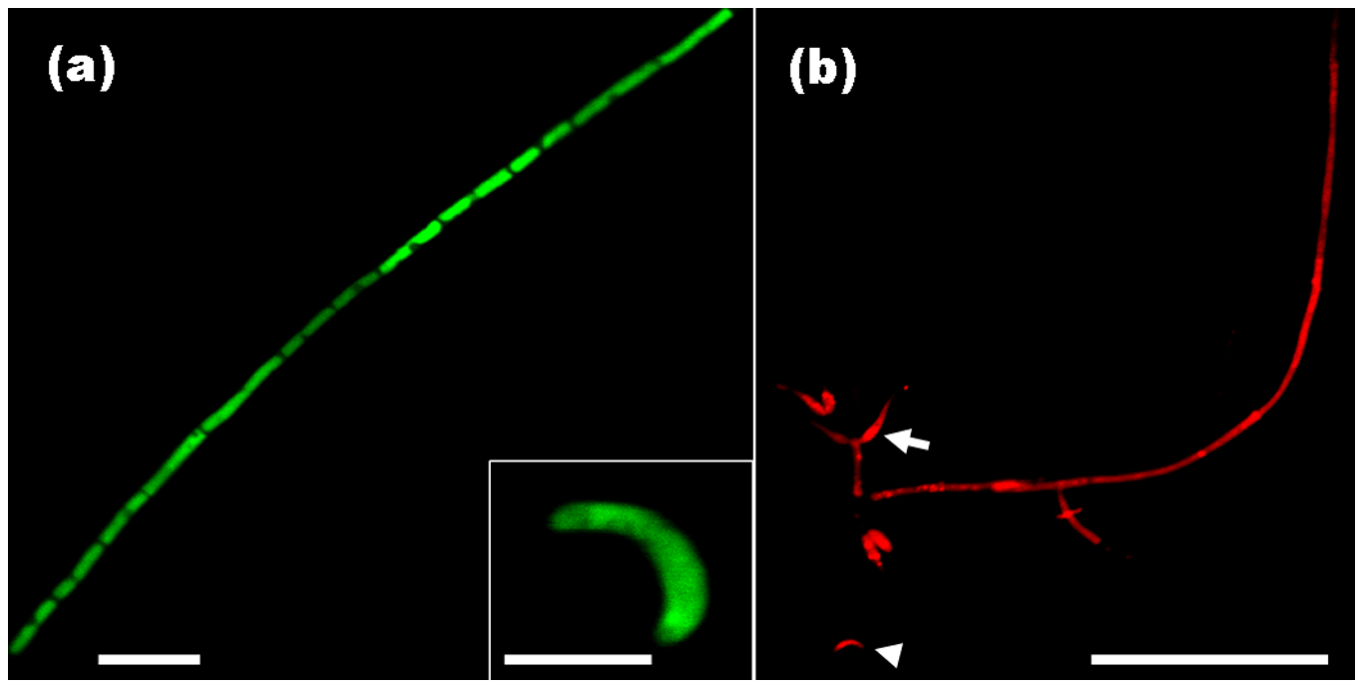
Laser scanning confocal microscopy of the *H. oryzae* transformants Ho31gfp and Ho19red. (a) Hyphae and falcate conidiophores of the Ho31gfp transformant showing constitutive eGFP expression. Bar, 20 µm; Bar in inner panel, 5 µm. (b) Hyphae, conidiophores (arrowhead) and flask-shaped phialides (arrow) of the Ho19red transformant showing strong red fluorescence. Bar, 50 µm.

After five generations, intense red and green fluorescence were found to be uniformly distributed within the hyphae, conidiophores and phialides of transformants Ho19red and Ho31gfp, respectively (Fig. 1). Ho19red and Ho31gfp were selected as candidates for further root inoculation.

### Dynamic Development of *H. oryzae* in Roots

**Figure 2:**
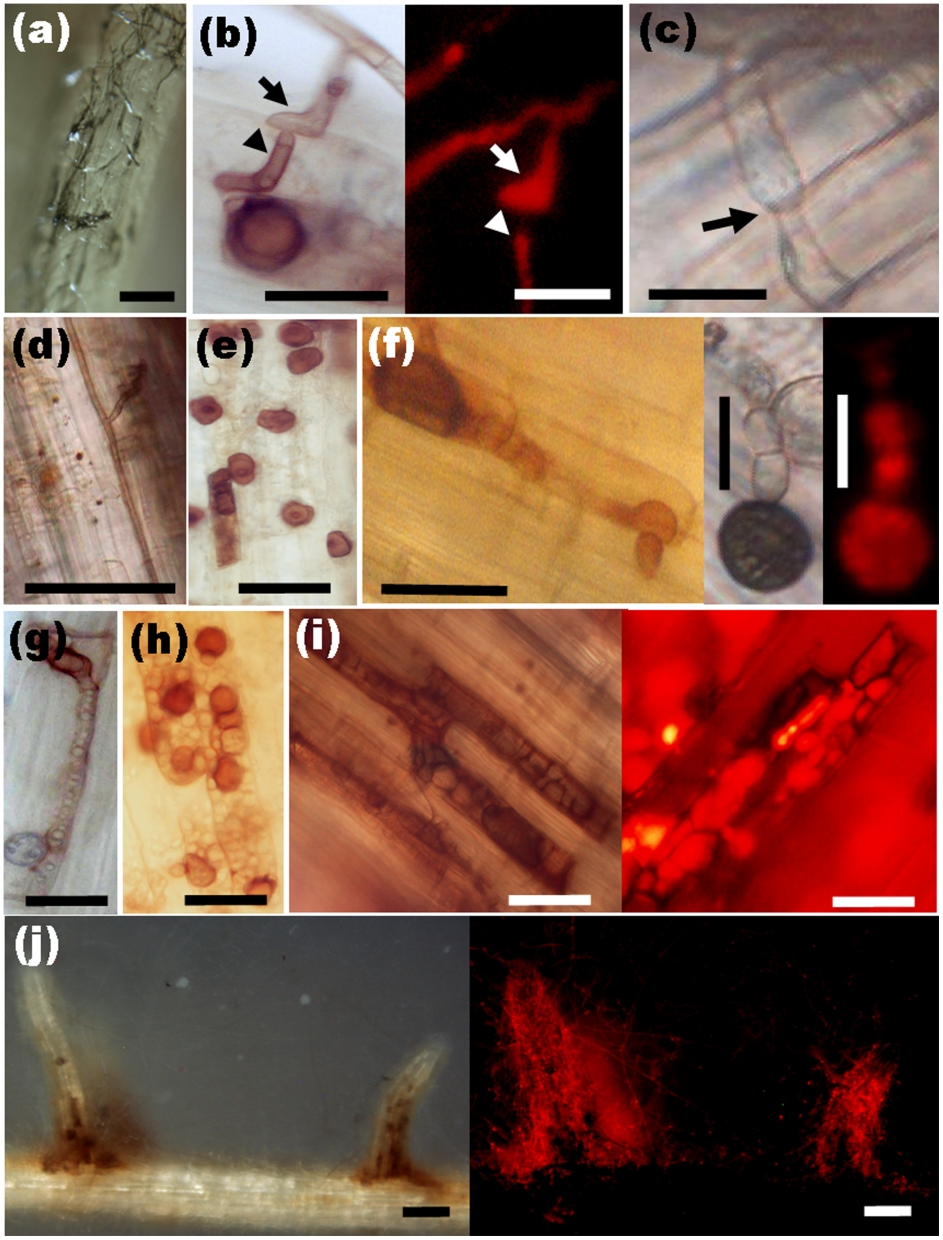
Fungal structures of *H. oryzae* during root infestation. (a) Dark runner hyphae enwrapping the root surface. Bar, 100 µm. (b) Light and fluorescence microscopy of hyphopodia (arrow) formed by runner hyphae as infection structures on the root surface and corresponding penetration peg (arrowhead). Bars, 20 µm. (c) Intracellular hyphae in cortical cells forming narrow neck-like constriction (arrow) where they cross the cell wall. Bar, 20 µm. (d) Intercellular hyphae expanding and branching occasionally in the cortical layer. Bar, 50 µm. (e) Darkly pigmented and thick-walled chlamydospores on the root surface. Bar, 20 µm. (f) Upon germination, chlamydospores producing septate germ tubes and appressorium-like bulges with corresponding infection pegs. Bars, 10 µm. (g) Germination of intracellular chlamydospores within epidermal cells. Bar, 20 µm. (h) Clusters of inflated, rounded, thick-walled cells compacted in the cortical cells before the formation of microsclerotia. Bar, 20 µm. (i) Light and fluorescence microscopy of darkly pigmented, irregularly lobed, thick-walled intracellular microsclerotia in the cortical cells. Bars, 50 µm. (j) Abundant hyphae and chlamydospores assembled at the basal parts of root hair cells. Bars, 500 µm.

**Figure 3:**
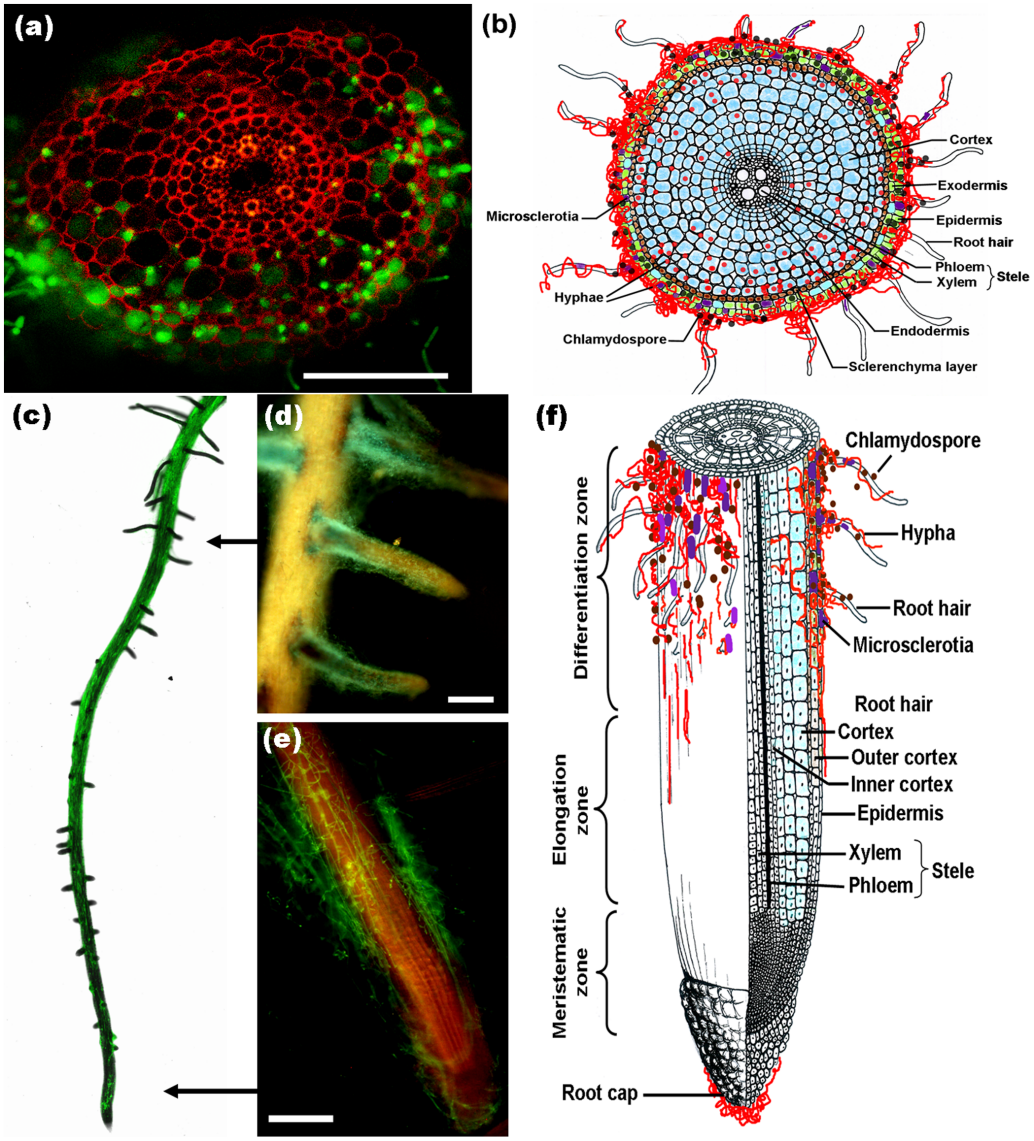
Colonization pattern of *H. oryzae* in rice roots. (a) In a root cross-section, eGFP-tagged hyphae gradually extended from the epidermis to the cortex without penetrating the stele. Bar, 200 µm. (c) A gradual increase in fungal colonization was associated with root maturation. Fungal colonization showing heavy colonization in the differentiation zone (d), slight colonization in the elongation zone, and no colonization in the meristematic zone (e). Bars, 500 µm. (b) and (f) Schematic representations of root colonization by *H. oryzae*. (b) The colonization pattern as seen in a transverse section. (f) Longitudinal section showing the association of fungal colonization with root maturation. Blue and green indicate living and dead cells, respectively. Red lines and dots: hyphae; black dots: chlamydospores; purple patches: microsclerotia.

Endophytic development and root colonization were monitored periodically. Root colonization was initiated from conidia, which formed dark “runner hyphae” upon germination (Fig. 2a) following the grooves between the epidermal cells on the root surface. Runner hyphae formed appressorium-like infection structures hyphopodia (Fig. 2b, arrow) on the surface and penetrated the epidermal cells via penetration pegs (Fig. 2b, arrowhead). Fungal growth was visible in the epidermal and cortical cell layers of the root. The intracellular hyphae were thick, with neck-like constrictions where they crossed the plant cell walls (Fig. 2c, arrow). The intercellular hyphae expanded parallel to the main axis of the root and occasionally branched into the intercellular spaces between cell walls (Fig. 2d). In addition to runner hyphae, darkly pigmented pearl-shaped chlamydospores formed on the root surface (Fig. 2e) germinated with septate tubes and developed swollen structures (Fig. 2f). The intracellular chlamydospores germinated and then completely filled a single cell before invading adjacent cells (Fig. 2g). During intracellular colonization, clusters of inflated, chlamydospore-like, thick-walled cells (Fig. 2h) were compacted within the cortical cells, which seemed to be the prophase of microsclerotia. When observing DSEs colonization, it is necessary to allow adequate time (> 15 d.a.i.) for the development of intracellular microsclerotia (Fig. 2i), which are described as compact darkly pigmented irregularly lobed thick-walled hyphae [5]. Later, fungal hyphae excessively occupied the epidermal and cortical cells, especially at the basal parts of the root hairs, in which branching hyphae formed a large number of chlamydospores and microsclerotia (Fig. 2j). Transversely, the fungus entered the root epidermis and later invaded from the outer cortex to the inner cortex (Fig. 3a, b). No hyphae approached the central part of the roots, resulting in their absence from the aerial parts of the plants. Concomitantly, a gradual increase of fungal colonization and proliferation generally associated with root maturation was observed (Fig. 3c, f). The root cap was slightly encompassed by hyphae, the root tip meristem zone showed no colonization, and the elongation zone showed mainly epidermal colonization with a few hyphae (Fig. 3e). In contrast, the differentiation zone was heavily occupied by intercellular and intracellular hyphae (Fig. 3d). Furthermore, fungal colonization did not harm the morphological development of the roots except that the colonized roots became melanized macroscopically (Fig. S1).

### Quantification of the *H. oryzae* Biomass in Root Tissue

**Figure 4:**
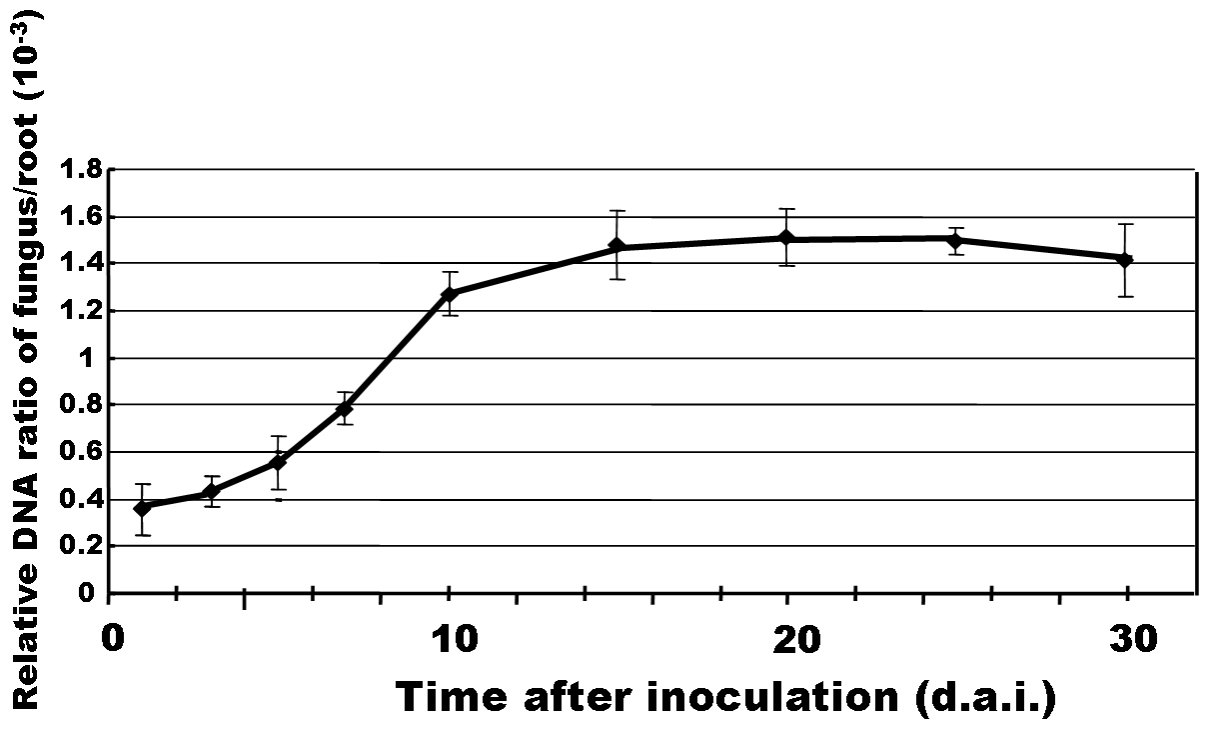
Relative amounts of fungal DNA in rice roots at different time points (1, 3, 5, 7, 10, 15, 20, 25 and 30 d.a.i.). A fungal colonization curve plotted with the means ± SD of six replicates is shown.

The FPDR was measured over time to allow the simultaneous assessment of fungal growth and the respective plant response. The results showed that an early moderate increase in the FPDR from 0.37×10^−3^ to 0.56×10^−3^ occurred within 5 d.a.i., followed by a significant increase to 1.28×10^−3^ at 10 d.a.i. and a final steady state at 1.49×10^−3^ from 15 d.a.i. onward (Fig. 4).

### Biotrophic Lifestyle of *H. oryzae* Accompanied by Host Cell Death

**Figure 5:**
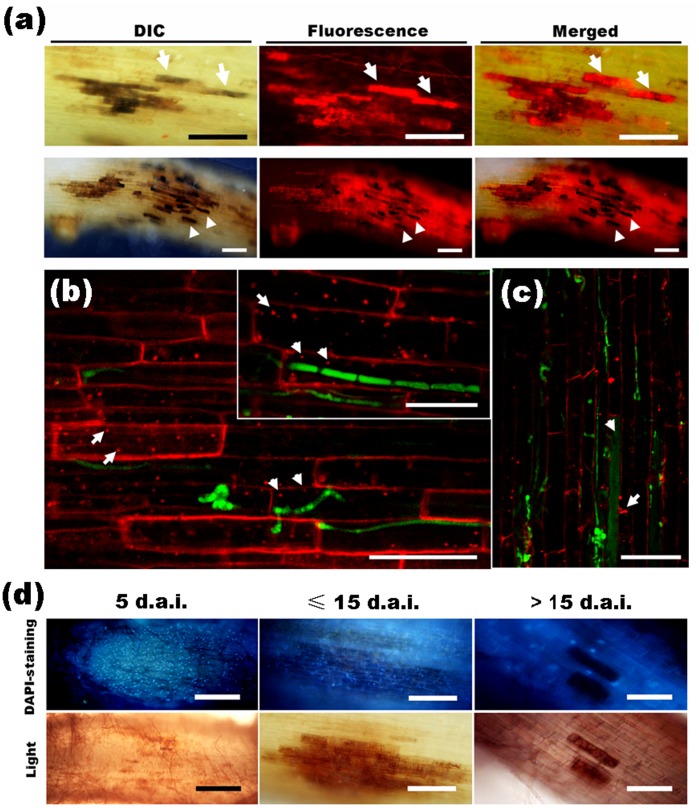
Vitality of *H. oryzae*-infected root cells as shown by staining with FM4-64 and DAPI, respectively. (a) The colonized cells became melanized lesions and showed enhanced fluorescence (arrows) from the intracellular hyphae at 10 d.a.i. (upper panels). Bars, 50 µm. Later (≥ 15 d.a.i.), additional hyphae penetrated the root cells and the lesion-like infected area became larger; moreover, the enhanced fluorescence and plant autofluorescence of some primary infected cells disappeared (arrowheads) (lower panels). Bars, 200 µm. (b) The internalization of FM4-64 into endomembrane structures in fungus-infected cells (arrowheads) and non-invaded cells (arrows) during early colonization (≤ 10 d.a.i.). Bars, 100 µm. (c) At 15 d.a.i., endocytosis disappeared in the cells occupied by microsclerotia (arrowhead), but remained in the adjacent non-infected cells (arrow). Bar, 50 µm. (d) Root segments stained with DAPI. A large number of DAPI-positive nuclei in root cells with slight infection at 5 d.a.i. (left panels; Bars, 400 µm). DAPI-stained nuclei in root cells occupied by abundant hyphae and microsclerotia at 15 d.a.i. (middle panels; Bars, 200 µm). DAPI-stained nuclei disappeared in root cells filled with microsclerotia, but remained in the adjacent non-invaded cells after 15 d.a.i. (right panels; Bars, 100 µm).

During early fungal colonization, the colonized cells became melanized like necrotic lesions in which enhanced red fluorescence (Fig. 5a, arrows) was emitted from the intracellular DsRed2-tagged fungal hyphae, indicating that the intracellular hyphae might remain alive. However, this enhanced fluorescence in partial cells, as well as the autofluorescence from the plant, later disappeared (Fig. 5a, arrowheads), speculating that these cells and the hyphae inside them might have been dead. Therefore, we investigated root cell vitality by FM4-64 staining. During early colonization (≤ 10 d.a.i.), *H. oryzae* hyphae penetrated and occupied root cells, in which internalization was observed in endomembrane structures (Fig. 5b, arrowheads) that formed in a similar fashion in non-invaded cells (Fig. 5b, arrows), indicating that fungal colonization did not affect cellular membrane dynamics or viability. However, in the late colonization stage (≥ 15 d.a.i.), in infected cells filled with plentiful hyphae and microsclerotia, endocytosis disappeared as well as endosome formation (Fig. 5c, arrowhead), indicating the occurrence of host cell death. Whereas the adjacent root cells remained alive, as proved by the endosome formation (Fig. 5c, arrow). Biotrophic colonization by *H. oryzae* was further substantiated by DAPI staining to visualize nuclei. At 5 d.a.i., root cells enwrapped by extensive runner hyphae and occupied by minor infection hyphae showed DAPI-positive nuclei (Fig. 5d), indicating that the root cells were alive. Subsequently, additional hyphae colonized the root cells to form microsclerotia less than 15 d.a.i. (Fig. 5d), the nuclei of these infected cells were still stained by DAPI, indicating that the colonized cells remained alive. From 15 d.a.i. onward, the fluorescence of the DAPI-stained nuclei gradually vanished while the intracellular microsclerotia formed (Fig. 5d), suggesting that cell death had taken place. It is noteworthy that the adjacent root cells remained alive, as demonstrated by the maintenance of DAPI-positive nuclei, even though the primary colonized cells were dead (Fig. 5d). In addition, in transverse root sections, almost all epidermal cells and outer cortical cells with heavy fungal colonization were dead, as reflected by the absence of DAPI-positive nuclei and plant autofluorescence (Fig. S2, arrowheads), whereas the inner cortical cells, which were occupied by relatively fewer intracellular and intercellular hyphae, remained vital with DAPI-positive nuclei (Fig. S2, arrows). Therefore, *H. oryzae* adopted an intimate biotrophic relationship with the root cells accompanied by partial host cell death.

### *H. oryzae*-induced Local Resistance against Root Infection by *M. oryzae*

**Figure 6:**
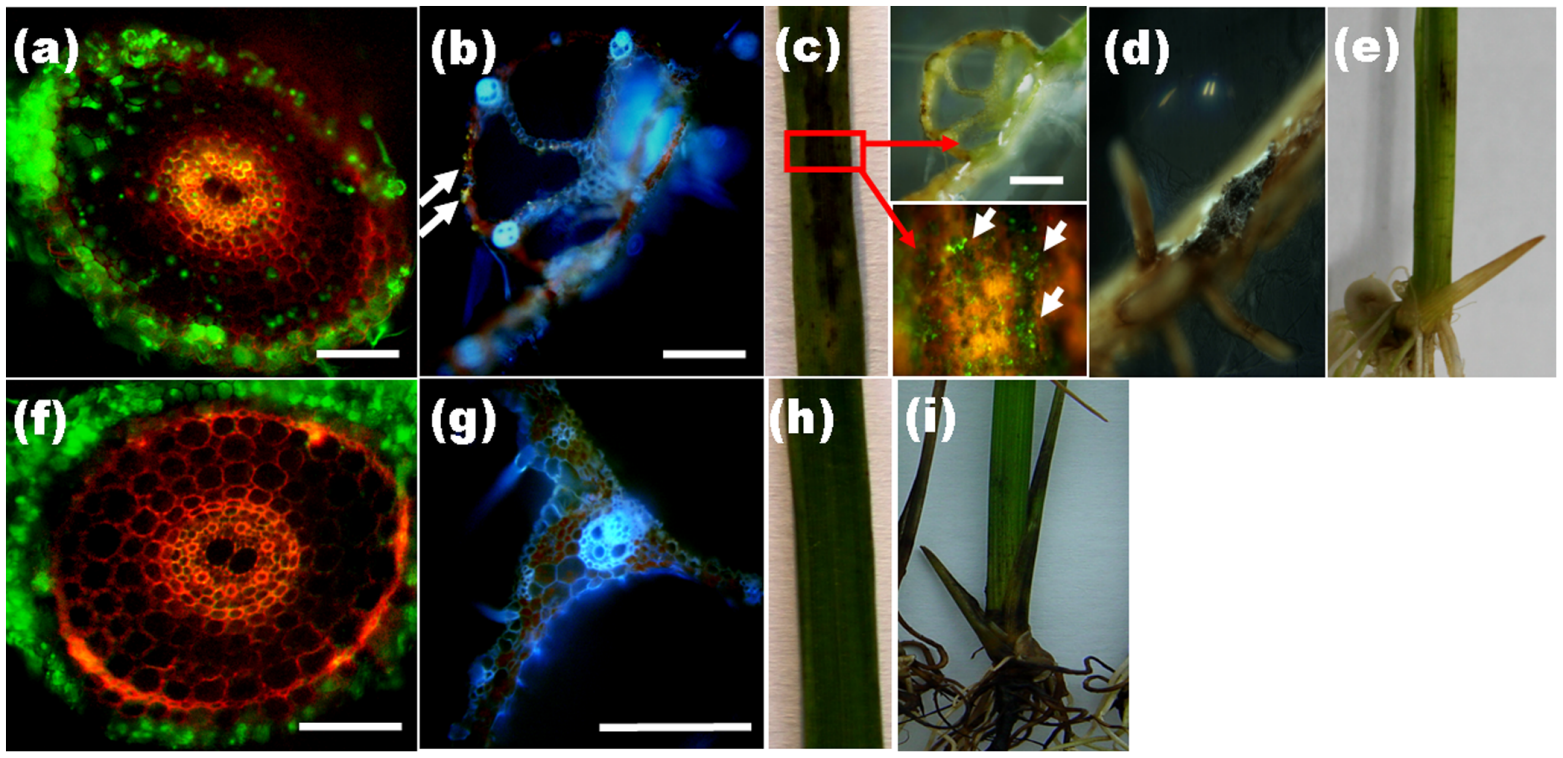
Effects of *H. oryzae* on root infection by *M. oryzae*. (a) – (e) In mock-infected roots, eGFP-tagged *M. oryzae* propagated in the stele (a) and spread systemically from root to leaf through the vascular tissue (b), causing typical blast symptoms in the leaves (c), roots (d) and stem (e). (f) – (i) In *H. oryzae*-infected roots, no eGFP-tagged *M. oryzae* hyphae emerged in the roots (f) and leaf vascular tissues (g), with the disappearance of blast disease on the leaves (h) and stem (i). Bars, 200 µm. Arrows indicate eGFP-tagged *M. oryzae*.

We investigated whether root colonization by *H. oryzae* would protect rice from infection by pathogens using *M. oryzae* as a test case. R5-6-1*-*infested plants were found to be more resistant to pathogen root invasion. Once inside the mock-inoculated root, eGFP-tagged *M. oryzae* was highly invasive and propagated from the cortical cells through the epidermis and into the stele (Fig. 6a). Furthermore, *M. oryzae* was seen to spread from the roots of rice plants to the aerial tissues (Fig. 6b), causing lesions on the leaves (Fig. 6c), roots (Fig. 6d) and diamond-shaped necrotic lesions at the base of the stem (Fig. 6e). In contrast, *M. oryzae* was not found in R5-6-1-inoculated roots (Fig. 6f) or aerial tissues (Fig. 6g), and the devastating effect of *M. oryzae* was markedly abolished with few necrotic lesions on the leaves and stems (Fig. 6h, i), suggesting that *M. oryzae* was unable to infect the roots of rice that had been co-cultured with R5-6-1 for 15 days. Similar to R5-6-1, inoculation with RC-3-1 showed an equivalent capacity to protect rice from *M. oryzae* root infection. These observations indicated that root colonization by *H. oryzae* had a significantly positive effect in protecting rice from pathogen root invasion.

**Figure 7:**
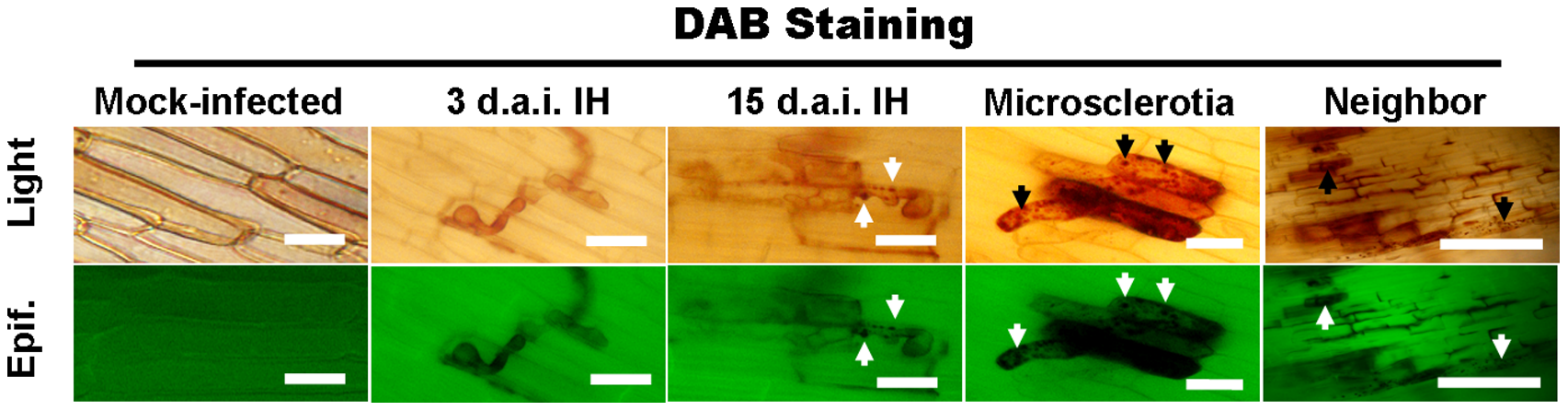
H_2_O_2_ accumulation caused by *H. oryzae*. Light and fluorescence microscopy of mock- and *H. oryzae*-infected rice roots at different time points after inoculation. The brown granules (arrows, DAB staining) indicate H_2_O_2_ accumulation. Bars, rightmost panels, 100 µm; others, 30 µm.

To investigate the reasons for this protective effect, the accumulation of H_2_O_2_ at sites of infection by *H. oryzae* was analyzed by staining with DAB at 3 d.a.i. (i.e., in the early stage of the plant-microorganism interaction), when hyphae proliferated in cortical cells through the epidermis after recognition, and at 15 d.a.i. (i.e., the late interaction stage), when specialized bulbous biotrophic infectious hyphae (IH) developed and intracellular dark microsclerotia were formed. R5-6-1-infected root cells containing IH and microsclerotia showed strong staining by DAB with the appearance of scattered brown granules along the IH and abundant granules along the microsclerotia, while fungus-infected cells at 3 d.a.i. and mock-infected cells showed no staining with DAB (Fig. 7). These observations indicated that the gradual accumulation of H_2_O_2_, an important type of host-driven ROS, at the sites of infection was induced by R5-6-1 in the late interaction period, and was related to the fungal load. The H_2_O_2_ accumulation level seemed to be proportional to the developmental stage and amount of hyphae because the level of H_2_O_2_ accumulation was lower in cells containing IH compared to those filled with extensive hyphae and microsclerotia (Fig. 7). Moreover, surrounding the primary infected cells, non-infected neighboring cells were also heavily stained with DAB, especially in the cell walls (Fig. 7), indicating that ROS diffused from the primary infected cells to neighboring cells or were directly induced in neighboring cells to reinforce large-scale resistance against secondary invasion by other microbes.

### Increased Antioxidant Capacity of *H. oryzae*-infested Roots

The beneficial protective effect of *H. oryzae* against *M. oryzae* root infection prompted us to investigate whether the antioxidant status of the infested roots was altered by the endophyte. R5-6-1-inoculated roots showed 12-fold greater SOD activity in comparison with controls (*P*<0.01). Meanwhile, CAT and POD activity was elevated by 52.1- and 39.8-fold after infestation with R5-6-1, respectively (*P*<0.01). As a major antioxidant buffer and free radical scavenger, the AsA level was enhanced slightly by 1.5-fold (*P*<0.05) after R5-6-1 inoculation. DHAR activity increased significantly, reaching a level 2.7-fold higher in R5-6-1-inoculated roots compared to controls. Concomitantly, we also found significantly enhanced total GSH concentrations and GR activity reaching 2.0- and 2.2-fold higher level after R5-6-1 inoculation, respectively (Table 1). Thus, colonization by *H. oryzae* increased the antioxidant levels and enzyme activities in rice roots.

### Systemic Resistance Induced by *H. oryzae*

**Figure 8:**
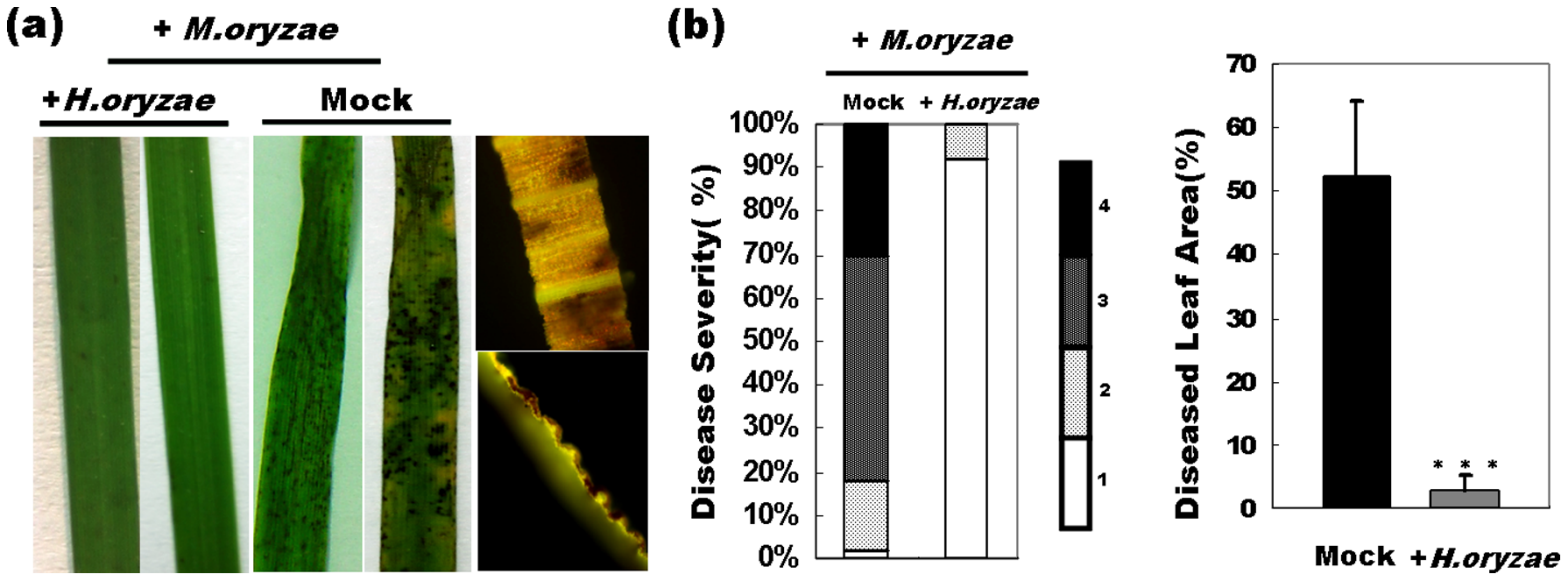
Systemic protection against rice blast by *H. oryzae*. (a) The alleviation of devastating symptoms on the leaves of *H. oryzae*-infected rice in contrast to mock-infected controls was observed. (b) Left panel, lesion severity was judged in four levels ranging from 1 (resistant) to 4 (highly susceptible) (each treatment, *n* = 50). Bars indicate the percentage of lesions with each severity level. Right panel, the lesion area was assessed by the %DLA using an Axiovision image analyzer. The values are the means ± SD from 50 leaves of *H. oryzae*-infected or mock-infected rice (***, *P*<0.001).

Having established local resistance, we also examined whether *H. oryzae* would trigger a systemic defensive response in rice. Therefore, we examined the consequences of R5-6-1 colonization upon foliar infection by *M. oryzae*. Marked reductions in disease lesion areas and severity were observed in R5-6-1-infected plants compared to controls (*P*<0.001) (Fig. 8b). These results indicated that disease lesions were restricted to a few tiny spots in R5-6-1-infested plants after *M. oryzae* spray inoculation, in contrast to the susceptible-type, spreading lesions in mock-infested plants (Fig. 8a). The disease area was assessed by monitoring the %DLA. The %DLA of the mock-infected plants was 52.3 ± 12%, whereas that of R5-6-1-infested plants was extremely low, only 3 ± 2% (Fig. 8b). Similarly, another *H. oryzae* strain, RC-3-1 attenuated *M. oryzae* foliar infection, exhibiting a coordinative management on *M. oryzae* like R5-6-1. Therefore, we concluded that colonization by *H. oryzae* indeed induced systemic resistance against rice blast.

Expression analysis revealed universal suppression in the expression of all PR genes with the exception of a negligible increase in *PR4* expression in R5-6-1-infested roots compared to controls (Table 2), suggesting that they were not dominant factors in the defense response. Moreover, the expression patterns of other foliar defense genes during R5-6-1 root infection resembled those of *M. oryzae*, showing biotrophy-accompanied defense suppression (Table 2).

**Table 2:** Expression of genes representative for plant defense response.

Gene Name	Description	TIGR	Fold change			
			1 h	3 h	1 d	3 d
***NAC4***	Transcription factor	Os01g60020.1	1.11	1.36	0.89	0.66
***CEBiP***	Chitin receptor	Os03g04110	0.52	0.53	0.36	0.22
***SK2***	Shikimate kinase	Os06g12150	1.76	0.84	1.04	0.93
***ORK10***	Ser/Thr protein kinase	Os01g02300	0.60	0.67	0.04	4.26
***Chit1***	Chitinase	Os02g39330	1.76	0.72	0.33	0.70
***OsWRKY53***	Transcription factor	Os05g27730	0.65	0.51	0.60	0.86
***OsWRKY71***	Transcription factor	Os02g08440	12.06	5.39	2.74	32.56
***JAmyb***	Transcription factor	Os02g08440	0.30	0.10	0.92	1.47
***NH1***	Ortholog of NPR1	Os01g09800	2.96	3.12	1.20	0.59
***PR1a***	Pathogenesis-related	Os07g03710	0.83	3.41	0.26	0.48
***PR2***	Pathogenesis-related	Os01g51570	0.01	0.03	0.005	0.11
***PR4***	Pathogenesis-related	Os11g37930	1.03	2.65	0.76	1.37
***PBZ1***	Pathogenesis-related	Os12t05551	1.09	2.03	0.35	0.31
***PR10b***	Pathogenesis-related	Os04g50710.1	0.70	1.49	0.31	0.26
***PR1b***	Pathogenesis-related	Os01g28450	0.57	0.82	0.55	0.31

**Figure 9:**
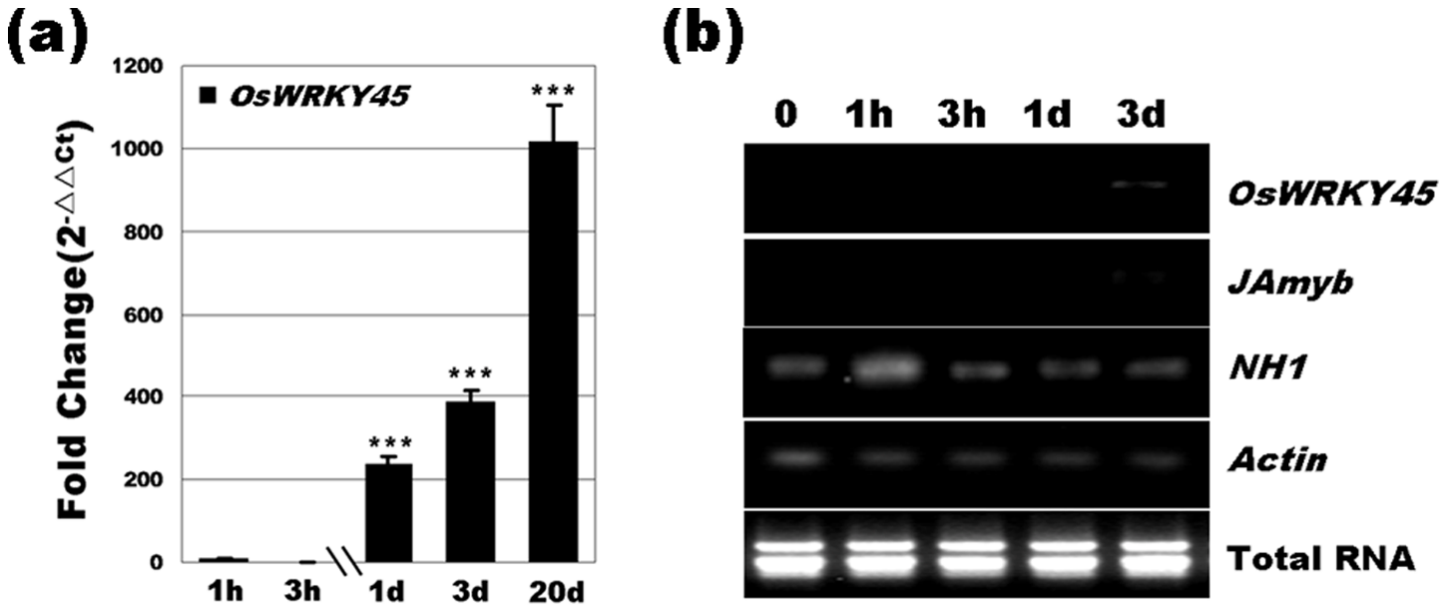
Temporal expression analysis of defense-related genes after *H. oryzae* inoculation. (a) *OsWRKY45* expression was markedly upregulated. (b) Analysis of *OsWRKY45*, *JAmyb* and *NH1* expression, which is indicative of different defense pathways. Bars represent the means (three replicates) ± SD. Significant differences (one-way ANOVA): *, *P*<0.05; **, *P*<0.01; ***, *P*<0.001.

In addition, the expression of *JAmyb* showed initial suppression and subsequent restoration to the original level (Table 2; Fig. 9b), suggesting that R5-6-1-induced systemic resistance was independent of JA. It should be noted that *OsWRKY45* expression was upregulated by 389-fold in R5-6-1-infested rice plants compared to mock-infected controls at 3 d.a.i. and maintained high expression, reaching over 1000-fold at 20 d.a.i. (Fig. 9a, b). The transcript accumulation of *NH1* showed initial weak induction followed by marked attenuation (Table 2; Fig. 9b), further substantiating the suggestion that *H. oryzae*-induced systemic resistance was *OsWRKY45*-dependent but independent of *NH1*. These results indicated that *OsWRKY45* played a crucial and predominant role in *H. oryzae*-induced defense responses, resulting in strongly sustainable enhanced resistance to blast infection.

## Discussion

### Root Colonization of *H. oryzae* in Rice

The colonization pattern of *H. oryzae* showed marked coincidence with the typical characteristics of DSEs [47], consisting mainly of three consecutive spatiotemporal phases. First, extracellular colonization was initiated by runner hyphae predominantly at the basal part of root hairs, which were regarded as preferential sites, then extended to weave a loose fungal network on the root surface (≤3 d.a.i.). In addition, chlamydospores could initiate colonization, which was slightly discrepant compared to *P. fortinii*, described as a typical representative DSE, with initial colonization characterized by superficial hyphae [47]. Next, it progressed to a biotrophic proliferation phase with intercellular and intracellular hyphae growing along the main axis of the root with centripetal branching and subsequent reproduction of extracellular and intracellular chlamydospores (<10 d.a.i.), while the colonized epidermal and cortical cells remained alive. Finally, cell death-associated colonization was observed, in which most epidermal and partial cortical cells inhabited by plentiful hyphae and microsclerotia in response to halting of further penetration underwent cell death (≥15 d.a.i.), while less infected adjacent cells remained alive. These observations indicated a close association between host cell death and abundant fungal hyphae. Longitudinally, fungal colonization increased gradually with tissue maturation, which was shown in *Piriformospora indica*-barley symbiosis [30]. Transversely, hyphae penetrated the cortex through the epidermis, reaching the inner cortex without deeper expansion into the stele, consistent with the typical root local colonization pattern of DSEs. Aside from the accordance to DSEs, this pattern was similar to those of other root endophytic fungi, including the soil-inhabiting fungi *Fusarium equiseti* and *Pochonia chlamydosporia* [35]. Due to their phylogenetically close relationship–both *H. oryzae* and *M. oryzae* belong to the Magnaporthaceae [17], [48], it is noteworthy that they formed strikingly similar fungal structures during root infection, including hyphopodia, runner hyphae and microsclerotia [49]. These comparisons suggest that regardless of whether the invader was endophytic or pathogenic, they would form similar fungal structures during root infection, which was associated with the type of infected tissue.

Nevertheless, there were some differences between *H. oryzae* and *M. oryzae*. These differences may be pivotal factors in determining whether the interaction pattern is mutualistic or antagonistic. The greatest difference was that *M. oryzae* exhibited strictly intracellular invasion and rapidly colonized vascular tissues [49], by which systemic infection to leaves and stems commenced [50], whereas *H. oryzae* failed to penetrate vascular tissues and showed local colonization in the roots. Although DSEs sometimes colonize the vascular cylinder in asymptomatic interactions, such colonization is frequently associated with pathogenicity [51], [52]. Therefore, whether fungi adopt non-restricted overexpansion or restricted expansion depends on their ability to occupy vascular tissue, which appears to be an essential determinant of antagonistic or mutualistic fungus-plant interactions. Furthermore, in *H. oryzae*-rice symbiosis, the fungus initially showed moderate proliferation, simultaneously inducing distinct root growth (reflected in a slow increase in the FPDR from 1 to 5 d.a.i.). Subsequently, the FPDR increased rapidly, suggesting more hyphae colonized within the roots, finally reaching a steady-state level in rice roots, indicating that fungal proliferation was synchronized with root growth. The fungal biomass was restricted to a certain extent without the ingression of additional hyphae from outside or overgrowth of the hyphae inside. This indicates a transition from an unlimited to a limited fungal growth pattern in the host root and homeostasis between root growth and fungal proliferation. In contrast, the pathogen showed unlimited proliferation in roots and even spread to aerial tissues accompanied by a continuous increase in fungal biomass. This fungal overgrowth led to plant disease and killed the host [53]. Therefore, another difference between mutualism and antagonism is quantitative rather than qualitative. Taken together, these observations indicate that colonization pattern and quantity are deciding factors in whether a fungus-plant interaction is mutualistic or antagonistic.

### Regulation of ROS in Plant-endophyte Symbiosis

In the *H. oryzae*-rice interaction, the accumulation of abundant plant-generated H_2_O_2_ was observed in dead or dying epidermal cells colonized by excessive fungal hyphae, suggesting that PCD contributed to the accumulation of H_2_O_2_, which was triggered by abundant hyphae as a plant defense to kill the invader directly or to trap them indirectly in dead cells. Thus, H_2_O_2_ may play a pivotal role in regulating the antagonistic homeostasis of cell death and fungal proliferation to maintain the fungal level in an allowable range. *P. indica* was reported to interfere with PCD to form a mutualistic interaction with barley [30], and *H. oryzae* also required PCD to maintain a mutualistic association with rice. Tanaka *et al.* [54] demonstrated that ROS originating from a mutualistic endophyte are required to inactivate plant defense responses against the fungus, thereby maintaining mutualism. It seemed that superfluous hyphae were the stimulus for ROS production, which in turn resulted in the counter-restriction of fungal proliferation and biomass by PCD.

Considerably less H_2_O_2_ was generated as a result of the *M. oryzae*-rice pathogenic interaction, in contrast to the abundant accumulation in *H. oryzae*-rice symbiosis, suggesting that virulent pathogens have developed mechanisms for scavenging ROS [31], while the H_2_O_2_-degrading ability of *H. oryzae* was lost or weakened during its long-term coevolution with plants, resulting in high sensitivity to oxidative stress. However, *H. oryzae* possesses melanin as an adaptive countermeasure to tolerate oxidative stress. Melanin not only acts as a pathogenicity factor [55], but also acts as a shield against oxidative stress. This suggests a close relationship between melanization and oxygen resistance [56]. Therefore, it is speculated that melanin, as the foremost characteristic of DSEs, is an evolutionary adaptation of *H. oryzae* in plant roots and is favorable for the coexistence of plants and fungi.

### ROS, Increased Antioxidative Capacity and Disease Resistance

ROS play an important role in priming PCD and act as signaling molecules to enhance plant defenses. In *H. oryzae*-rice, due to the accumulation of H_2_O_2_, some epidermal and outer cortical cells underwent PCD, thereby blocking the invasion of biotrophic *M. oryzae* and restricting its access to water and nutrients. Such competition of preemptive resource utilization appeared to be one explanation for the enhanced local resistance induced by *H.oryzae* against *M. oryzae*. Moreover, as H_2_O_2_ is a diffusible molecule in biological membranes, it also acts as an intracellular signal to activate large-scale defense responses against subsequent infection by *M. oryzae*. Therefore, *H. oryzae* induced ROS leading to cell death and a lack of sites for *M. oryzae*, which played a crucial role in regulating the mutualistic interaction and enhanced disease resistance.

Antioxidants are the first line of defense against free radical and ROS damage, and are therefore critical for maintaining the optimum health of plant cells. In *H. oryzae*-rice symbiosis, the activities of SOD, CAT, POD, DHAR and GR were significantly enhanced due to the accumulation of H_2_O_2_. Meanwhile, the concentrations of AsA and GSH increased. In plant cells, antioxidant enzymes and metabolites act in synergy to detoxify ROS. Accumulating data indicate that plant resistance is due to an ability to increase the activity of ROS-detoxifying enzymes or the biosynthesis or regeneration of antioxidant metabolites [57]. Therefore, the increases in antioxidant enzymes and metabolites induced by *H. oryzae* helped strengthen local resistance against infestation by *M. oryzae*.

In the interplay between *H. oryzae* and rice, the activation of ROS and antioxidants may be the mechanism underlying the host resistance response.

### SA-mediated *OsWRKY45*-dependent and *NH1*-independent Systemic Resistance

In *H. oryzae-*rice, a transient initial induction and subsequent attenuation of defense-related gene expression were observed during the early stages. This suppression of defense genes is similar to that in other biotrophic fungal endophytes and pathogens, including *M. oryzae* [49], *Ustilago maydis* [58], *Blumeria graminis* [59] and *P. indica* [60]. These results confirm that the suppression of plant defensive responses is associated with the *H. oryzae* biotrophic lifestyle and is favorable for the establishment of *H. oryzae*-rice symbiosis. In addition, the suppression of plant-biotroph interactions may be related to recognition and penetration mechanisms and be common and conserved in mutualism and antagonism, although there were differences in both speed and magnitude.

SA, JA and ethylene (ET) are involved in regulating and inducing basal resistance against different pathogens. SA is a key regulator of pathogen-induced systemic acquired resistance, whereas JA and ET are required for rhizobacteria-mediated induced systemic resistance. Typically, SA-dependent signaling pathway is induced in response to biotrophic pathogens [61], [62]. In contrast, the JA/ET-dependent signaling pathway is typically activated in response to necrotrophic pathogens [63]. In rice-*H. oryzae* symbiosis, the expression of *JAmyb* which is involved in the JA-mediated, SA-independent signaling pathway in rice [64], was reduced, indicating that *H. oryzae*-induced systemic resistance is independent of the JA signaling pathway. However, *OsWRKY45*, which is an SA-mediated but *NH1*-independent defense signal in rice [65], was markedly upregulated with no concomitant rise in *OsNPR1* expression. As reported previously, the SA signaling pathway in rice branches into two subpathways: *OsNPR1*- and *OsWRKY45*-dependent pathways [65]. Therefore, the systemic resistance induced by *H. oryzae* against blast infection was mediated by *OsWRKY45*-dependent and *OsNPR1*-independent SA signaling pathway.

Interestingly, *OsWRKY45* expression was also upregulated when rice was inoculated with *M. oryzae*. Nevertheless, the upregulation of *OsWRKY45* caused by *M. oryzae* was much faster and sharper than that of *H. oryzae* (data not shown). Rice *OsWRKY45* plays a crucial and predominant role in BTH-inducible defense responses and thus strongly enhances resistance to blast infection [65], [66]. Therefore, it was speculated that *OsWRKY45*-dependent SA signaling pathway is a specific defense strategy in rice for *M. oryzae* and its close relative, *H. oryzae*, suggesting that an exclusive defense pathway is activated depending on the specific type of microbe and host. Although there are many similarities between these two closely related fungi, they showed distinct interaction patterns with rice. This raises the question of what triggers the switch from a mutualistic to an antagonistic interaction. Comparative transcriptomic and genomic analyses of these two interaction patterns (i.e., rice-*H. oryzae* and rice-*M. oryzae*) will provide novel insights and greater understanding of the molecular mechanisms and pivotal transcripts involved in the switch from mutualism to antagonism. The available rice and fungal mutants defective in signal transduction and reverse genetics will facilitate the elucidation of the molecular basis of symbiosis (be doing now).

### Importance of DSEs for Agricultural Management

The present study established a new symbiotic system between rice and *H. oryzae*. Based on the results presented here, the mutualistic symbiosis of crop plants and DSEs has great potential for sustainable agriculture. Both *H. oryzae* strains R5-6-1 and RC-3-1 from wild rice roots were capable of establishing mutualistic relationships in rice roots and producing a beneficial response with respect to the protection of aboveground and belowground tissues from invasion by the rice blast pathogen, *M. oryzae*. From a long-term agronomical viewpoint, *H. oryzae* promises to confer positive effects on disease resistance and cereal yield. The exploitation and utilization of DSEs such as *H. oryzae* may, therefore, not only curtail the input of fungicides and fertilizers in crop production but also may be a novel resource for improving both disease resistance and grain yield.

This study indicates that a wild rice-derived endophytic fungus exhibits a unique protective role against a destructive pathogen of cultivar rice. In addition, this mutualistic symbiosis provides a useful model for investigating the molecular mechanisms of growth promotion and protection from biotic and abiotic stresses.

## Supporting Information

Figure S1 **Melanized roots colonized by** ***H. oryzae*** **without any deformation in comparison with the controls.** Left, non-inoculated roots; right, *H. oryzae*-inoculated roots.
(TIF)

Figure S2 **Transversal section of DAPI-stained rice roots. DAPI-negative nuclei (arrowheads) were observed in the epidermal and outer cortical cells, while the cortical cells remained alive with DAPI-positive nuclei (arrows).** Bars, 200 µm.
(TIF)

Table S1 **Primers used for real-time PCR and qRT-PCR.**
(DOC)
