# Supplementary figures and images for: Evidence for Biotrophic Lifestyle and Biocontrol Potential of Dark Septate Endophyte Harpophora oryzae to Rice Blast Disease
Source: PLoS One. 2013 Apr 18;8(4):e61332. doi: 10.1371/journal.pone.0061332 (PMC3630206; doi:10.1371/journal.pone.0061332)

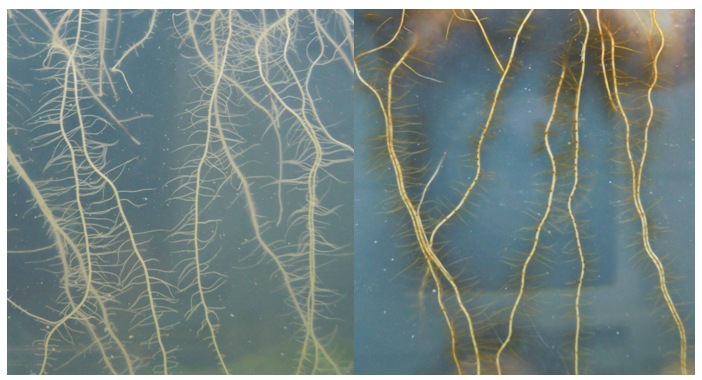

Supplement: Figure S1 — Melanized roots colonized by H. oryzae without any deformation in comparison with the controls. Left, non-inoculated roots; right, H. oryzae-inoculated roots. (TIF) [file pone.0061332.s001.tif]

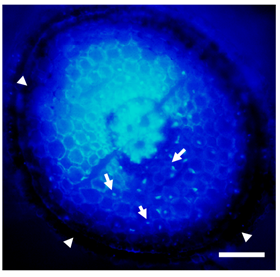

Supplement: Figure S2 — Transversal section of DAPI-stained rice roots. DAPI-negative nuclei (arrowheads) were observed in the epidermal and outer cortical cells, while the cortical cells remained alive with DAPI-positive nuclei (arrows). Bars, 200 µm. (TIF) [file pone.0061332.s002.tif]
