# Supplementary material for: Evidence for Biotrophic Lifestyle and Biocontrol Potential of Dark Septate Endophyte Harpophora oryzae to Rice Blast Disease
Source: PLoS One. 2013 Apr 18;8(4):e61332. doi: 10.1371/journal.pone.0061332 (PMC3630206; doi:10.1371/journal.pone.0061332)
Supplement: Table S1 — Primers used for real-time PCR and qRT-PCR. (DOC) [file pone.0061332.s003.doc]

Table S1. Primers for Real-time PCR and qRT-PCR

| Gene | Forward (5'-3') | Reverse (5'-3') |
| --- | --- | --- |
| *Tef* | CATCGAGAAGTTCGAGAAGG | TACTTGAAGGAACCCTTACC |
| SR | TTTTCTCCGACAATCCAGACC | AAAAGAGTGCTAGAACCCCG |
| *SK2* | GCTAAATTTTGCTTGTGTTCAGC | AACAGGAGGGCTTCATCAAC |
| *NAC4* | AAGCGCAGCATCAACAAAG | TCCATCCTTCTCCTCTCGTG |
| *ORK10* | GCAAACTAGGATAGCGACTTGA | TAACCCGCAAAAAGATGAGG |
| *Chit1* | CTGGTACTGGACCAACAACG | GTTCTTGCCGTCGCACTC |
| *CEBiP* | GATGACTGGTTTATCCAGCTTTG | TTCAAGCAGCCGTACAAGTG |
| *JAmyb* | CGACCATCGGCAATTTCATTCGGT | ACCGTTAAGCTGGTTGGTCCTGAA |
| *OsWRKY53* | CCAATTTGTTGATTCGTTGC | CGTACGCGTATCCCAAGTGT |
| *OsWRKY71* | AGATGGCGATGACGCTGAC | AGCAATCGTCAATCCTTGGT |
| *OsWRKY45* | CGGGTAAAACGATCGAAAGA | TTTCGAAAGCGGAAGAACAG |
| *PR10b* | TCTCCGTATTGCTGCTTCCT | CACTCTCACAAAATCAAACACCA |
| *PR4* | CCACATGGGATGCCAACAA | AGGCGGTCCATCCATACTTCT |
| *PR1a* | TCTTCATCACCTGCAACTACTC | ATTCATCGGATTTATTCTCACC |
| *PBZ1* | CTACTATGGCATGCTCAAGAT | ATAGAAAGGCACATAAACACAA |
| *PR-2* | AAGATTGTTCTGAGAAGAGATCGATCGA | GCTACGCGAAAATAGGTCTGGTAAACTT |
| *OsNPR1* | TGGCAGGTGAGAGTCTACGA | AGGTGGATTTGCACCAGAAC |
| *OsUbiq* | GTGGTGGCCAGTAAGTCCTC | GGACACAATGATTAGGGATCA |
| *Actin* | CTTCAACACCCCTGCTATG | CCGTTGTGGTGAATGAGTAA |
